# The role of AI in reducing maternal mortality: Current impacts and future potentials: Protocol for an analytical cross-sectional study

**DOI:** 10.1371/journal.pone.0323533

**Published:** 2025-05-14

**Authors:** Patrick O. Owoche, Morris Senghor Shisanya, Betty Mayeku, Lucy Natecho Namusonge

**Affiliations:** 1 School of Computing and Informatics, Kibabii University, Bungoma, Kenya; 2 School of Nursing, Kibabii University, Bungoma, Kenya; University of Ilorin, NIGERIA

## Abstract

**Background:**

Maternal and newborn mortality remains a critical public health challenge, particularly in resource-limited settings. Despite global efforts, Kenya continues to report high maternal mortality rates of over 350 deaths per 100,000 live births and a neonatal mortality rate of 21 per 1,000 live births. Artificial Intelligence (AI)-enabled maternal healthcare interventions, such as Obstetric Point-of-Care Ultrasound (OPOCUS) and AI-driven SMS intervention on Promoting Mothers through Pregnancy and Postpartum (PROMPTS), offer innovative solutions to improve early detection, diagnosis, and maternal health-seeking behaviors. However, there is limited evidence on their usability, feasibility, and impact on maternal and neonatal outcomes.

**Objective:**

This study aims to assess the implementation, user experiences, and impact of OPOCUS and PROMPTS on maternal and neonatal health outcomes in Kenya. Specifically, it evaluates their effectiveness in reducing maternal complications, improving antenatal and postnatal care utilization, and enhancing clinical decision-making while identifying potential barriers to adoption and scalability.

**Methods:**

This mixed-methods, cross-sectional study will be conducted in ten counties in Kenya that have integrated AI-based maternal healthcare interventions. Quantitative data will be collected from health facility records, national health databases (KHIS), and structured surveys, while qualitative data will be gathered through key informant interviews (KIIs) with healthcare providers and policymakers, as well as focus group discussions (FGDs) with maternal health service users. Statistical analyses will include comparative pre- and post-AI implementation assessments, with thematic analysis for qualitative insights.

**Expected outcomes:**

The study will generate empirical evidence on the feasibility, effectiveness, and barriers to AI integration in maternal health services. Findings will inform policy recommendations, enhance AI-assisted maternal healthcare design, and support the scaling of AI-driven interventions to improve maternal and neonatal health outcomes in Kenya and other low-resource settings.

**Conclusion:**

AI-based maternal health interventions hold promise for reducing maternal mortality, improving diagnostic accuracy, and enhancing health-seeking behaviors. However, their success depends on user experiences, healthcare system readiness, and policy alignment. This study will provide critical insights for evidence-based scaling and policy integration of AI in maternal healthcare.

## Introduction

High maternal and newborn mortality and morbidity remains a pressing problem globally and more so in developing countries [1]. In 2020, the global Maternal Mortality Ratio (MMR) was estimated at 223 maternal deaths per 100,000 live births, showing a decline from 227 in 2015 and 339 in 2000 [2]. Despite significant improvements, maternal mortality remains a disaster in the African Region, where the MMR was 531 maternal deaths per 100,000 live births in 2020. The African Region accounts for more than two-thirds (69%) of maternal deaths globally, signalling an urgent need for intensified efforts to reduce MMR to 70 maternal deaths per 100,000 live births by 2030 to meet the Sustainable Development Goals (SDGs) [3]. An estimated 500,000 women and 3 million newborns die annually from preventable pregnancy and childbirth-related complications [2]. Major complications accounting for nearly 75% of maternal deaths include severe hemorrhage (25%), infection (15%), high blood pressure during pregnancy (12%), obstructed labor (8%), and unsafe abortion (13%) [1,3].

In Kenya, maternal and newborn mortality rates are still alarmingly high, with over 350 maternal deaths per 100,000 live births and a neonatal mortality rate of 21 per 1,000 live births. While these rates are below the Sub-Saharan average, progress in maternal and neonatal health in Kenya remains slow [4]. Efforts to improve maternal health are hindered by inadequate healthcare infrastructure, shortages of skilled healthcare providers, and insufficient access to essential medicines and comprehensive emergency obstetric care services.

The quality of maternity care is critical in preventing maternal and newborn mortality. Timely interventions for conditions such as postpartum hemorrhage, eclampsia, obstructed labor, and sepsis are essential. Many maternal and early newborn deaths can be prevented through the adoption of Artificial Intelligence (AI) [5]. AI can significantly enhance maternal and newborn healthcare by improving early detection and diagnosis, providing personalized care, enabling remote monitoring and telemedicine, supporting clinical decision-making, optimizing healthcare resource allocation, and facilitating continuous learning and improvement [6]. Obstetric Point-of-Care Ultrasound (OPOCUS) has improved maternal and neonatal outcomes through enhanced diagnostic accuracy and early detection of pregnancy complications. AI-driven image processing detects fetal growth restrictions and placental abnormalities, enabling timely interventions [7]. Additionally, predictive models assess risks like preterm birth and gestational diabetes, facilitating personalized care [8]. In resource-limited areas, AI-powered handheld ultrasounds improve prenatal care access by addressing cost and training barriers [9].

AI-driven SMS interventions like PROMPTS enhance maternal health by increasing antenatal visits, postnatal check-ups, and health awareness [10,11]. Machine learning optimizes pregnancy care, improving perinatal outcomes and preterm birth prevention [12]. The PROMPTS platform, part of the Kuboresha Afya Mitaani (KAM) study, provided stage-specific messaging and clinical support via SMS, significantly improving care-seeking behaviors among 1,416 mothers in Nairobi’s informal settlements [13].

While integrating AI like OPOCUS and PROMPTS into maternal healthcare contributes to safer pregnancies, better health outcomes, and equitable access to quality care, studies on user experiences and outcomes of OPOCUS and PROMPTS are essential to bridge evidence gaps, assess feasibility, evaluate impact, and inform policy [14]. Understanding healthcare providers’ experiences with OPOCUS and maternal engagement with PROMPTS will optimize their design, training, and integration into routine care. Additionally, assessing their impact on maternal and neonatal outcomes will provide evidence for scaling and policy alignment, ensuring AI-powered solutions contribute effectively to universal health coverage and maternal health equity [15,16].

### Problem statement

Despite advancements in maternal healthcare, preventable maternal and newborn deaths remain a significant challenge, particularly in resource-limited settings. Timely detection and intervention for complications such as postpartum hemorrhage, eclampsia, obstructed labor, and sepsis are crucial in reducing maternal and neonatal mortality. Artificial Intelligence (AI)-enabled tools like OPOCUS) and PROMPTS have shown promise in enhancing pregnancy care by improving diagnostic accuracy, risk assessment, remote monitoring, and patient engagement [17–19]. OPOCUS facilitates early detection of fetal growth restrictions and placental abnormalities, enabling timely interventions, while PROMPTS improves maternal health-seeking behaviors and access to essential information. However, there is limited evidence on user experiences, feasibility, and the real-world impact of these AI-powered solutions on maternal and neonatal outcomes [20]. Understanding how healthcare providers interact with OPOCUS and how pregnant and postnatal women engage with PROMPTS is essential for optimizing their effectiveness, addressing implementation barriers, and ensuring equitable healthcare access. Without evidence on their usability, adoption, and integration into routine maternal care, scaling these innovations remains uncertain. This study aims to bridge this gap by evaluating user experiences and health outcomes associated with OPOCUS and PROMPTS, providing critical insights for evidence-based scaling, policy formulation, and AI-driven improvements in maternal healthcare.

### Study aim

In this study, we aim to assess the current use of AI technologies in maternal healthcare, specifically focusing on Obstetric Point-of-Care Ultrasound (OPOCUS) and AI-driven SMS intervention, PROMPTS. By examining how these AI tools are integrated into maternal health services, we will evaluate their usability, accessibility, and effectiveness in clinical settings. Furthermore, this study seeks to evaluate the impact of AI on maternal health outcomes, with a particular emphasis on reducing maternal mortality and improving prenatal and postnatal care. Through a comparative analysis of maternal health indicators before and after AI implementation, we will determine whether these technologies contribute to earlier detection of complications, improved care-seeking behaviors, and better health outcomes for both mothers and newborns. Additionally, this research will explore the perceived future potentials and barriers to AI adoption in maternal healthcare. By gathering insights from healthcare providers, policy makers, and maternal health clients, we will identify key challenges such as infrastructure limitations, workforce readiness, ethical concerns, and policy gaps that may hinder the scaling of AI interventions. At the same time, we will examine opportunities for expanding AI-driven maternal healthcare solutions to ensure equitable, effective, and sustainable improvements in maternal and newborn health outcomes. As we address these objectives, the study will provide critical evidence to inform policy, guide AI integration strategies, and enhance maternal healthcare delivery in Kenya and similar resource-limited settings.

### Justification

The integration of AI technologies in maternal and newborn healthcare offers a transformative approach to reducing maternal mortality and improving health outcomes. AI-driven innovations like OPOCUS and PROMPTS enhance early diagnosis, risk assessment, and personalized care, addressing critical gaps in timely interventions and healthcare accessibility. However, despite their potential, evidence on their real-world impact, user experiences, and implementation challenges remains limited, particularly in low- and middle-income countries (LMICs). Understanding the feasibility, effectiveness, and integration of these AI solutions is essential for ensuring their scalability and sustainability.

This study will provide empirical evidence on how AI-enabled interventions improve maternal healthcare delivery while identifying barriers to adoption, ethical considerations, and policy implications. By combining quantitative health outcomes with qualitative user feedback, the study will offer actionable insights for optimizing AI-driven maternal health solutions, supporting equitable healthcare access, and guiding future policy and implementation strategies. The findings will contribute to global efforts in reducing maternal mortality and ensuring AI’s responsible and effective deployment in maternal healthcare systems..

## Methodology

### Study site and research design

This study employs a mixed-methods approach that combines both quantitative and qualitative data collection and analysis to comprehensively assess the impact of AI tools on maternal healthcare outcomes in healthcare facilities in ten counties in Kenya namely Bungoma, Kakamega, Kisumu, Nakuru, Kiambu, Nairobi, Kitui, Kilifi, Mombasa, and Kwale that are implementing OPOCUS and PROMPTS AI technologies in reversing adverse trends in maternal health care. This study selects ten counties based on their implementation of OPOCUS and PROMPTS. These counties represent a mix of urban, peri-urban, and rural settings, ensuring geographic and demographic diversity. They were chosen due to their high maternal mortality burden, health system readiness for AI integration, and ongoing digital health initiatives.

### Target population

This study will focus on healthcare professionals, policymakers, and maternal health service users to comprehensively assess the implementation, impact, and future prospects of AI-enabled maternal healthcare interventions such as Obstetric Point-of-Care Ultrasound (OPOCUS) and AI-driven SMS interventions like PROMPTS.

### Sample size and sampling methodology

The sample size for this study on will be estimated using Cochrane’s formula for infinite population. This approach will ensure that the sample is representative and adequate to draw meaningful conclusions.

### Sample size estimation

The sample size of clients will be determined based on the number of deliveries conducted in 2023 in the 10 counties (671839 live births). Cochrane’s formula for infinite population was used where $n_{0}=(Z^{2}.p.(1-p))/e^{2}$. Where Z = 1.96 (for 95% confidence level), p = 0.262 (estimated population proportion of deliveries for 2023 as projected by Ministry of Health and Kenya National Bureau of Statistics) [21,22] and e = 0.03 (3% margin of error). Thus n_0_=(3.8416*0.193356)/0.0009 825.33 thus 826 plus 10% error and nonresponse is 83 = 909 for OPOCUS and for PROMPTS each.

### Sampling

A sampling frame will be developed using patient outpatient numbers or inpatient numbers. Systematic sampling will be employed to ensure every nth patient from the list is selected, ensuring a random and representative sample. N^th^ value will be attained by applying the applying sample to population proportion (909/671839 = 0.00135) to the number of clients on the sampling frame, i.e., if the sampling frame has n number of clients then N^th^ value will be 0.00135*n.

All health policy makers involved in reproductive health (RH) at the national and county levels will be included in the study. This comprehensive inclusion ensures that all relevant perspectives and policies are considered.

All implementers of AI-assisted maternal health services in the selected facilities will be interviewed. This includes healthcare workers directly involved in the application and management of AI technologies in maternal health.

### Inclusion exclusion criteria

#### Inclusion criteria.

Clients will include women aged 18 years and above who have given birth within the specified study periods, both pre- and post-AI implementation. These women must be residents of the regions where AI-assisted maternal health services are implemented and have utilized these services at the respective facilities. Additionally, they must be willing and able to provide informed consent to participate in the study.

Health Policy Makers included in the study will be those involved in reproductive health policy-making at the national and county levels. They must have at least one year of experience in health policy development or implementation related to maternal health and be willing to participate in the study by providing informed consent.

Implementers in Facilities will comprise healthcare providers and staff directly involved in the implementation and management of AI-assisted maternal health services. These participants must have at least six months of experience working with AI technologies in maternal health and be willing to participate in the study and provide informed consent.

#### Exclusion criteria.

The exclusion criteria are set to eliminate individuals who may not provide relevant data or whose participation might be impaired.

Clients excluded from the study will be women under 18 years of age, non-residents of the regions where AI-assisted maternal health services are implemented, and those who have not utilized these services at the designated facilities. Additionally, women who are unwilling or unable to provide informed consent or who have severe mental or physical conditions impairing their ability to participate in interviews or complete questionnaires will also be excluded.

Health Policy Makers excluded from the study will be those not directly involved in reproductive health policy-making at the national or county levels, those with less than one year of experience in health policy development or implementation related to maternal health, and those unwilling to participate or provide informed consent.

Implementers in Facilities excluded from the study will be healthcare providers and staff not involved in the implementation and management of AI-assisted maternal health services, those with less than six months of experience working with AI technologies in maternal health, and those unwilling to participate or provide informed consent.

### Data collection

Data collection is anticipated to start on 20^th^ August 2024–3^rd^ March 2025 and will follow a sequential mixed-methods approach, beginning with qualitative data collection to inform and enhance the subsequent quantitative phase. Initially, in-depth interviews and focus group discussions with healthcare providers, patients, and policy makers will be conducted to gather detailed insights and identify key themes, challenges, and barriers to OPOCUS and PROMPTS integration in maternal health. These qualitative findings will be meticulously analyzed to refine and develop more precise and relevant quantitative survey instruments. By incorporating the deeper understanding gained from qualitative data, the quantitative tools will be more robust, context-specific, and comprehensive, ensuring they effectively capture the critical variables and metrics needed for a thorough analysis. This iterative process aims to create a more accurate and holistic understanding of AI’s impact on maternal health outcomes.

### Qualitative data collection

#### Policy analysis and stakeholder engagement.

This policy analysis aims to evaluate the regulatory framework, strategic vision, and implementation challenges associated with AI-enabled maternal healthcare interventions (OPOCUS and PROMPTS). The analysis will focus on policy gaps, ethical considerations, infrastructure needs, and stakeholder engagement to inform evidence-based AI integration in maternal health.

1 **Review of policy and regulatory framework**

A systematic review of policy documents, guidelines, strategic plans, and reports from the Ministry of Health, county health departments, and relevant agencies will be conducted. The document review will focus on 4:

- Existing AI policies and compliance with international maternal health standards.
- Strategic vision and leadership commitment to AI integration.
- Resource allocation and infrastructure support for AI initiatives.
- Stakeholder engagement and multi-sectoral collaboration in AI policy-making.
- Ethical, legal, and social considerations in AI-driven maternal healthcare.
- Monitoring and evaluation (M&E) frameworks for AI-driven maternal health interventions.

An initial list of relevant documents will be compiled through literature review and key informant recommendations, supplemented by reference tracking and additional sources identified during interviews.

2 **Key informant interviews (KIIs)**
  A **Policy makers and health administrators**

KIIs will be conducted with policy makers at the national and county levels, including:

- Senior officials from the Ministry of Health (MoH).
- County health department representatives.
- Leaders from NGOs and multilateral health organizations.

Themes explored during interviews:

1. AI policies and guidelines in maternal healthcare.
2. Strategic vision for AI adoption in the health sector.
3. Resource allocation and infrastructure support for AI initiatives.
4. Stakeholder engagement in AI policy formulation.
5. Ethical and social considerations in AI use for maternal health.
6. M&E frameworks for AI interventions in maternal care.
7. Challenges and barriers to AI implementation.
8. Future policy directions for AI in maternal and newborn healthcare.

Interview process:

- Conducted at Ministry of Health (Afya House) and select counties.
- Semi-structured format with two public health experts as interviewers (ensuring gender representation).

B **Healthcare workers in AI-integrated facilities**

KIIs with healthcare providers implementing AI-assisted maternal health services will assess real-world adoption, usability, and challenges of OPOCUS and PROMPTS.

Themes explored:

1. Usability and workflow integration of AI technologies.
2. Impact of AI interventions on maternal health outcomes.
3. Training and support provided for AI technology adoption.
4. Challenges and barriers to AI implementation.
5. Patient feedback and satisfaction with AI-assisted maternal care.
6. Recommendations for improving AI integration into maternal health services.

Sampling approach:

- Purposive sampling to ensure representation from public hospitals, private clinics, and maternal health NGOs.

3 **Focus Group Discussions (FGDs) with Mothers Using AI-Enhanced Maternal Health Services**

FGDs will be conducted with pregnant and postnatal women who have engaged with AI-driven maternal health services (OPOCUS and PROMPTS). Discussions will focus on accessibility, usability, impact on maternal health behaviors, and satisfaction.

Themes explored:

1. Access to AI-enhanced maternal health services.
2. Ease of use and understanding of AI-driven tools.
3. Impact of AI on early detection and management of pregnancy complications.
4. Trust and confidence in AI-assisted maternal care.
5. Comparisons between AI-enhanced and traditional maternal care.
6. Recommendations for improving AI-assisted services.

Sampling approach:

- Diverse representation of clients from urban and rural settings and different socioeconomic backgrounds.
- Trained moderators will facilitate discussions.

### Quantitative data collection

To ensure a comprehensive evaluation of AI-enabled maternal healthcare interventions, data will be collected from multiple sources, including health facility records, national health databases, and participant surveys. The study will employ both quantitative and qualitative methods to assess feasibility, implementation barriers, and the impact of OPOCUS and PROMPTS on maternal and neonatal outcomes.

#### Primary data sources.

1 **Health facility records**

- Data from hospitals and clinics implementing AI-assisted maternal health services, specifically those using OPOCUS and PROMPTS.
- Patient records, including prenatal care logs, ultrasound findings, delivery outcomes, and postnatal care follow-ups.
- AI intervention logs detailing usage patterns, diagnostic outputs, and clinical recommendations.

2 **National and regional health databases**

- Kenya Health Information System (KHIS) and Ministry of Health reports on maternal health indicators.
- Aggregate data on prenatal visits, delivery outcomes, and maternal mortality trends in AI-implemented facilities.

3 **Surveys and interviews**

- Healthcare provider surveys & interviews to assess experiences with OPOCUS, its usability, perceived diagnostic accuracy, integration into routine care, and barriers to adoption.
- Maternal surveys & focus group discussions for gathering insights from antenatal and postnatal mothers on their engagement with PROMPTS, perceived benefits, impact on health-seeking behaviors, and challenges in adoption.

#### Data collection variables.

The key variables as as summarized below and in Table 1.

**Table 1:** Summary of key variables.

Variable Type	Variable Name	Measurement
**Independent** **Variables**	OPOCUS usage	Yes/No, Frequency, Diagnostic Impact
	PROMPTS usage	Yes/No, Frequency, Health Messages Received
	Healthcare provider engagement with AI	Yes/No, Training, Usability
	Policy support for AI	Policy presence, Funding allocation
**Dependent** **Variables**	Maternal Mortality Rate	Deaths per 100,000 live births
	Maternal complications	Occurrence of hemorrhage, eclampsia, sepsis
	Prenatal care utilization	Number of ANC visits, Timing of first ANC
	Delivery outcomes	Mode of delivery, Presence of complications
	Neonatal outcomes	Birth weight, Apgar score, Neonatal mortality
	AI acceptability	Likert scales on ease of use, trust in AI
**Moderating/** **Confounding Variables**	Maternal age, Education level, Socioeconomic status	Categorical (Low, Medium, High)
	Health system infrastructure	Facility type, Provider availability
	Cultural factors	AI literacy, Traditional health-seeking behavior

1 **Demographic data**

Collected for all participants to analyze factors influencing maternal health outcomes:

- Maternal age, education level, marital status, occupation, socioeconomic status, and geographic location (urban/rural).

2 **Prenatal care utilization (Pre- and post-AI implementation)**

- Number of antenatal visits.
- Gestational age at first visit.
- Types of screenings conducted (e.g., ultrasound via OPOCUS, blood pressure, glucose tests).
- Interventions received (e.g., iron supplementation, tetanus toxoid).

3 **AI-enabled interventions (OPOCUS & PROMPTS usage patterns)**

- Frequency of OPOCUS use per patient.
- AI-detected complications (e.g., fetal growth restriction, placental abnormalities).
- Clinical actions taken based on OPOCUS findings.
- Frequency and content of PROMPTS messages received.
- Patient-reported adherence to recommended health actions prompted by AI-driven SMS.

4 **Delivery outcomes**

- Mode of delivery (vaginal vs. Caesarean or assisted/ instrumental).
- Presence of complications (e.g., hemorrhage, obstructed labor, hypertensive disorders).
- Neonatal outcomes (birth weight, Apgar scores, neonatal mortality).

5 **Maternal health outcomes**

- Maternal complications during pregnancy and postpartum.
- Maternal mortality trends (collected from hospital and national records).
- Changes in causes of maternal mortality before and after AI intervention.

6 **Health-Seeking Behaviors & Patient Engagement**

- Change in frequency of antenatal and postnatal visits after exposure to PROMPTS.
- Self-reported adherence to health advice provided via AI-driven SMS.
- Patient satisfaction and perceived impact of AI interventions on care access and quality.

### Data analysis

#### 1. Qualitative data analysis.

The qualitative component will focus on user experiences, feasibility, and barriers related to the integration of OPOCUS and PROMPTS into maternal healthcare.

***Thematic analysis approach*.** Data will be analyzed thematically to identify key patterns and insights on:

1. Usability and workflow integration of OPOCUS.
2. Impact of AI-driven SMS interventions on maternal health-seeking behaviors.
3. Perceived effectiveness of AI-enabled maternal care.
4. Challenges and barriers to AI adoption (cost, training, system integration).
5. Ethical and policy considerations in AI-driven maternal care.

Findings will inform and refine the quantitative survey instruments to ensure alignment with real-world experiences.

#### 2. Quantitative data analysis.

The quantitative analysis will focus on maternal health indicators, AI utilization trends, and impact assessment.

***Data sources and cleaning***

- Data will be gathered from hospital and clinic records, national databases (KHIS), and structured surveys.
- Data cleaning and validation will include cross-referencing records, handling missing data, and removing inconsistencies.

***Statistical analysis methods***

1 **Descriptive analysis**

- Summarize key maternal health indicators before and after AI intervention.
- Examine trends in prenatal visits, delivery outcomes, maternal complications, and neonatal health.

2 **Comparative analysis (pre- vs. post-AI implementation)**

- T-tests and Chi-square tests will compare maternal health outcomes before and after OPOCUS and PROMPTS integration.
- Odds ratios (ORs) and confidence intervals (CIs) will be used to assess significant differences in outcomes.

3 **Impact assessment of AI interventions**

- Differences in maternal health outcomes between those who received OPOCUS/PROMPTS and those who did not.
- Key indicators for comparison:
  1. Prenatal care utilization (ANC visits, early screenings).
  2. Skilled birth attendance and delivery outcomes.
  3. Newborn care interventions (early neonatal check-ups, care for at-risk newborns).
  4. Lead time in diagnosing complications (trimester of complication detection).
  5. Postnatal care utilization and immunization rates.
- Multivariate regression models may be applied to control for confounders such as maternal age, socioeconomic status, and geographic location.

#### 3. Integration of qualitative and quantitative findings.

A mixed-methods approach will be used to provide a comprehensive evaluation of AI’s impact on maternal healthcare.

- Qualitative insights will contextualize quantitative trends, explaining variations in AI adoption, usability, and health-seeking behaviors.
- Integration will focus on:
  - Why OPOCUS and PROMPTS improve (or do not improve) outcomes based on user experiences.
  - Barriers to AI adoption that might explain disparities in quantitative results.
  - Policy implications for scaling AI-driven maternal health interventions in Kenya.

By synthesizing statistical trends and lived experiences, this study will generate actionable insights for evidence-based decision-making on AI in maternal healthcare.

### Ethical considerations

The proposed research project will carefully consider ethical issues at each stage of its implementation, especially those involving human subjects. Approval for this study has been sought from Jaramogi Oginga Odinga University of Science and Technology (JOOUST) Institutional Ethics Review Committee (IERC). Additionally, an official data collection permission letter will be obtained from the County Directors of Health. Research authorization and a permit will be acquired from the National Commission for Science, Technology, and Innovation (NACOSTI). Informed consent will be obtained from all participants after they have been introduced to the study and fully informed about their rights. To ensure confidentiality and privacy, participants’ names will not be recorded in the data abstraction forms, questionnaires or during Key informant interviews and Focus Group Discussions, and data collection will be conducted in private settings. The principle of justice and impartiality will be upheld by providing equal opportunities for all individuals in the target population to participate in the study through the use of probability sampling methods. This approach ensures that the selection process is fair and unbiased. The potential risks associated with participation are minimal, primarily involving possible discomfort during interviews or focus groups. However, the benefits include significant contributions to improving maternal healthcare through evidence-based AI interventions.

## Discussion

### Protocol rigor

The dedicated study core team for this cross-sectional study on AI integration in maternal health services in Kenya will consist of the Principal Investigator (PI) and two Co-Principal Investigators (Co-PIs). This core team will serve as the custodians of study-specific procedures, ensuring adherence to the research protocol and standard operating procedures to guarantee accurate data collection and high-quality results.

To maintain quality, consistency, and harmonized study procedures, standardized training, supervision, and oversight will be provided. This includes regular monitoring and support to ensure all team members understand and follow the established protocols.

The core team will oversee the following critical areas:

- Finalizing the study title and sourcing for funding.
- Managing protocol amendments and follow-ups on data collection.
- Ensuring proper data management and implementing recommendations from the data management team.
- Setting recruitment start and end dates.
- Monitoring actual recruitment rates versus projected rates.
- Managing the consenting process for participants.
- Summarizing and addressing protocol deviations.
- Conducting site visits and reporting any organizational issues or study-related problems.

The data management team will include the PI, Data Clerks at the study sites, the study nurse, and Community Health Volunteers (CHVs). Their responsibilities will encompass:

- Establishing the acceptance rate proportion, comparing those who accepted to participate with those sampled.
- Monitoring participant retention and completeness of data from recruitment through data collection.
- Providing monthly forecasts of data collection progress.
- Monitoring loss to follow-up as a proportion of participants who do not complete the study questionnaires or assessments.
- Overseeing data management metrics, such as the rate of electronic data capture, return dates, and the rate of returns.
- Tracking the number of completed surveys and questionnaires.
- Ensuring data fidelity through monitoring by the study nurse or CHV.
- Conducting data quality checks and reviewing results through a dashboard.

### Dissemination plans

Upon completion of data analysis, the results of this study will be utilized by the core research team to develop manuscripts for submission to peer-reviewed journals and presentations at international conferences. These efforts will specifically target professionals involved in maternal health service provision in low-resource settings, as well as policy makers and advisors in these areas. Additionally, a comprehensive report will be compiled by the PI and archived in the Kibabii University repository.

There will be a feedback session organized with healthcare professionals, both at operational and strategic levels, within the regions where the study was conducted. The findings will also be disseminated through popular media portals such as Twitter, Facebook, and local newspapers to reach a broader audience.

While the study protocols will be published in peer-reviewed journals, participant-level data sets and analysis code will be made available upon reasonable written request to ensure transparency and foster further research.

### Limitations of the study design

This cross-sectional study may be subject to selection bias, as it focuses on women attending ANC clinics and delivering in hospitals, limiting generalizability to those outside formal healthcare. Recall bias may affect self-reported adherence to AI-driven interventions like PROMPTS, while social desirability bias could influence responses on satisfaction and trust in AI technologies. To mitigate these, the study will ensure diverse representation, supplement self-reports with hospital records and AI usage logs, and use anonymous surveys and open-ended interviews. Despite these limitations, the study remains valid for assessing the feasibility, usability, and impact of AI-driven maternal healthcare interventions.

### Amendments to the study

The PI will be responsible for communicating any important protocol modifications, such as changes to eligibility criteria, outcomes, or analyses, to all relevant parties. This includes study implementers, the JOOUST Institutional Ethics Review Committee (IERC), the journal PLOS ONE, and the National Commission for Science, Technology and Innovation (NACOSTI).
